# Correlations between horse neck crest scoring and nape fat measurements obtained after image analysis

**DOI:** 10.1186/1751-0147-57-S1-P11

**Published:** 2015-09-25

**Authors:** Severiano R Silva, Rita Payan-Carreira, Cristina M Guedes, S Coelho, Ana Sofia Santos

**Affiliations:** 1 Animal and Veterinary Research Center, CECAV, Universidade de Trás-os-Montes e Alto Douro, Vila Real, Portugal; 2 Escola Universitária Vasco da Gama, Coimbra, Portugal

## Introduction

Obesity can be an important problem in companion horses and ponies and has been associated to an increased disease risk. Body condition scoring is the most common method to assess adiposity, though recently some improvements were introduced in adiposity evaluation in horses, like the neck crest adiposity rating. In horses neck crest adiposity was linked to insulin resistance and laminitis, but little is known about the relationship between the neck crest scores (NCS) and the thickness of the nape fat in carcasses.

## Objective

This study aimed to establish the correlation between nape fat measurements and NCS in horses.

## Material and methods

Prior to slaughter, two evaluators determined the NCS in 17horses, using a scale from 0 to 5. Images from the lateral view of hemi-carcasses were captured after slaughter using a digital camera, under standard artificial light and at a constant predetermined position of the camera relative to the carcasses. Eight megapixel images were analysed using ImageJ software to obtain the area, the major axis and minor axis of the nape fat. Correlation analysis was performed to obtain the relationship between NCS and nape fat measurements.

## Results

The correlation between NCS and nape fat measurements was 0.636, 0.676 and 0.791 (p<0.01) for the area, and for major and minor axis respectively.

## Conclusion

These results show a clear relationship between the NCS and the nape fat. Further studies with different breeds, gender and body condition are needed to fully understand the relationship between NCS and nape fat.

